# Prognosis value and positive association of Rab1A/IL4Rα aberrant expression in gastric cancer

**DOI:** 10.1038/s41598-023-33955-w

**Published:** 2023-04-28

**Authors:** Haoran Li, Zhengwu Cheng, Bin Jiang, Xinyu Shao, Menglin Xu

**Affiliations:** 1 Department of Gastrointestinal Surgery, The First Affiliated Hospital of Wannan Medical College, Wuhu, 241000 China; 2 Department of Hepatobiliary Surgery, The First Affiliated Hospital of Wannan Medical College, Wuhu, 241000 China; 3 Department of Gastroenterology, Suzhou Municipal Hospital, Affiliated Suzhou Hospital of Nanjing Medical University, No. 242 Guangji Road, Suzhou, 215006 Jiangsu China; 4 Department of Oncology, The First Affiliated Hospital of Wannan Medical College, No. 2 Zheshan West Road, Jinghu District, Wuhu, 241000 Anhui China

**Keywords:** Cancer, Molecular biology, Biomarkers, Oncology

## Abstract

Gastric cancer (GC) is the most common gastrointestinal cancer and the leading cause of worldwide cancer-associated mortality. Several GC patients are diagnosed at the advanced stage with an unsatisfactory 5-year survival rate. Rab1A was significantly associated with IL4Rα expression in non-small cell lung cancer. However, their potential correlation in expression and prognosis remains largely unknown in GC. In this study, Rab1A/IL-4Rα was significantly increased in GC than in para-cancerous tissues, and Rab1A/IL-4Rα overexpression caused poor prognosis among GC patients. Rab1A expression was significantly correlated with IL-4Rα expression in GC tissues, as determined by IHC analysis. In addition, the mRNA expression of Rab1A was closely linked with the IL-4Rα mRNA expression in GC tissue expressed by *qPCR*. Furthermore, the Kaplan–Meier analysis demonstrated that the group with negative Rab1A and IL-4Rα expression had longer 5-year survival rates than the other group. Besides, the group with positive Rab1A and IL-4Rα expression had a worse prognosis than the other group. Finally, nomograms revealed the overall 3 and 5-year survival determined crucial roles of Rab1A/IL-4Rα expression in predicting the prognosis of GC patients. Therefore, Rab1A/IL-4Rα is vital in GC, providing a novel perspective on targeted GC therapy.

## Introduction

Gastric cancer (GC) is the most common gastrointestinal cancer with the leading cause of cancer-associated global mortality^1,2^. Although diverse and systematic treatments have been employed for GC, including surgical treatment, chemoradiotherapy, and targeted therapy, the current 5-year survival rate remains unsatisfactory^3,4^. Unfortunately, most GC patients are diagnosed at an advanced stage, with distant organ metastasis, leading to poor prognosis^5^. Moreover, long-term use of chemotherapy drugs causes tumor cell drug resistance, and insensitivity to tumor drugs limits the chemotherapy efficacy^6^. Therefore, finding novel therapeutic targets to promote the efficacy of GC treatment is an urgent requirement.

Rab proteins are members of the GTP enzyme superfamily and are recognized as housekeeping proteins in intracellular membrane dynamics involving various membrane transport processes. Rab1A, a member of the Rab family^7^, is a GTP enzyme controlling vesicular transport from the ER to the Golgi apparatus^8,9^. Recent studies indicated Rab1A regulates signal transduction^10^, cell migration^11^, and cell autophagy^12^. Furthermore, abnormal Rab1A expression is associated with some clinical disease occurrences, including Parkinson's disease^13^ and primary cardiomyopathy^14^. Previous studies reported that Rab1A was aberrantly expressed in many cancers, leading to poor prognoses, including breast^15^, lung^16^, and liver^17^ cancers. Moreover, Rab1A behaved as an oncogene in various cancers, enhancing growth, metastasis, and cancer cell drug resistance^18,19^.

Interleukin-4 (IL-4) and its receptors (IL-4R) are crucial in cancer cell proliferation and other biological behaviors like migration and invasion^20,21^. Additionally, IL-4R promoted cancer cell resistance to chemotherapy drugs in colorectal cancer^22^. One of the receptor chains of IL-4Rα was highly expressed in solid cancers and was closely associated with locally advanced tumor staging, promoting poor prognosis^23,24^.

Recent research reported that Rab1A promotes non-small cell lung cancer metastasis by stabilizing the IL4Rα protein. Moreover, Rab1A was significantly correlated with IL4Rα expression, including the region and degree of expression^25^. However, the potential correlation of Rab1A with IL4Rα in expression and prognosis remains largely unknown in GC. This study mainly explores the correlation between Rab1A and IL4Rα expression and the effect of Rab1A/IL4Rα on the prognosis of GC patients.

## Results

### Expression levels of Rab1A in GC and paired adjacent tissues

**Figure 1:**
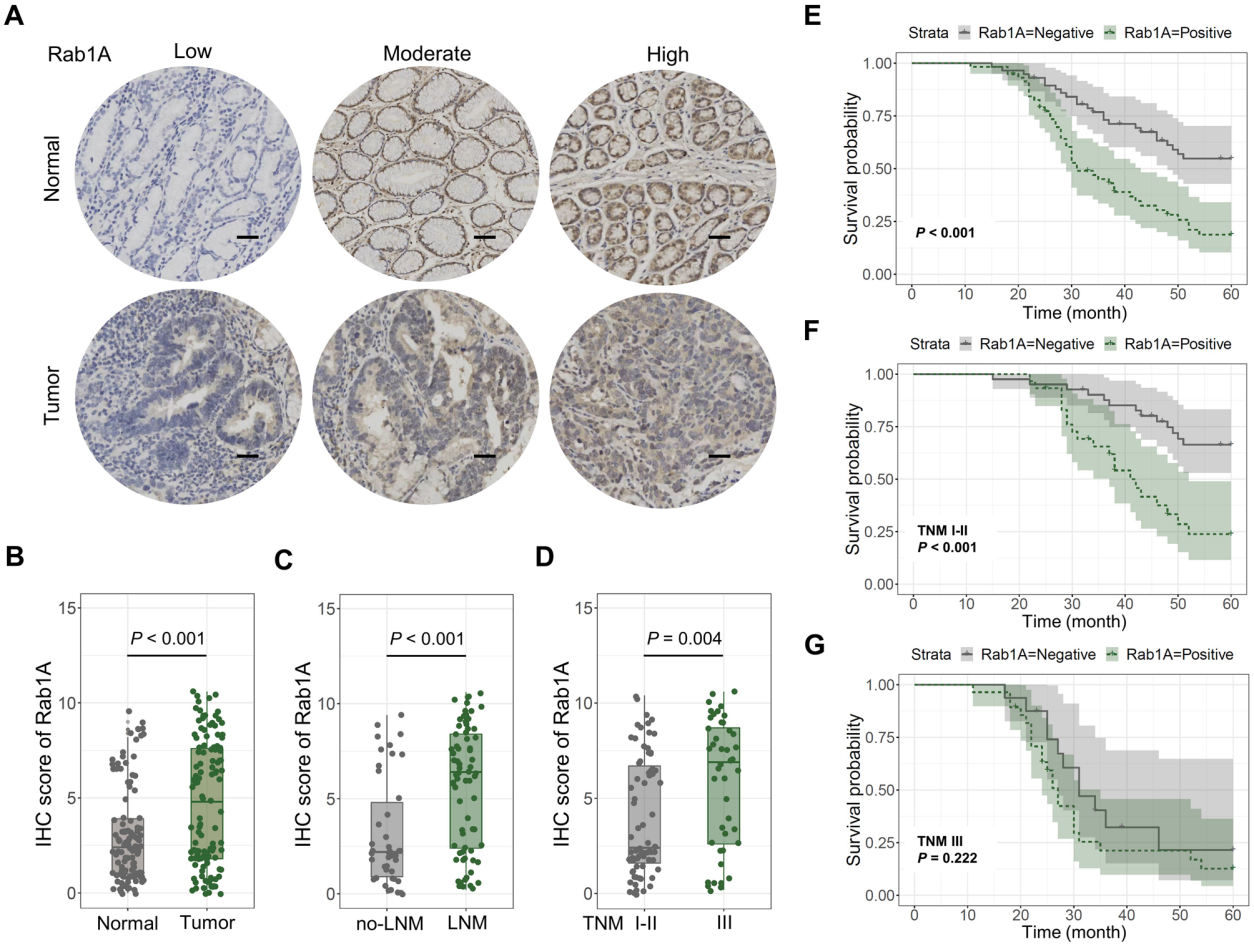
Rab1A expression and Kaplan–Meier analysis among 115 GC patients. (**A**) Rab1A expression using IHC staining in 115 GC and para-cancer tissues (scale bar 100um). (**B**–**D**) The IHC score of Rab1A in (**B**) GC and para-cancer tissues, (**C**) GC with or without lymph node metastasis, (**D**) GC with TNM I–II or TNM III staging. (**E**–**G**) Kaplan–Meier analysis of Rab1A positive vs. Rab1A negative in (**E**) 115 GC patients, (**F**) GC patients with TMN I–II staging, and (**G**) GC patients with TMN III staging.

Firstly, IHC staining helped investigate the Rab1A expression in 115 GC patients (Fig. 1A). The results indicated that Rab1A was significantly expressed in GC tissues than in matched normal ones (P < 0.001) (Fig. 1B). Moreover, subgroup analysis was performed based on the lymph node metastases (LNM) and tumor-lymph node-metastasis (TNM) staging state. Rab1A expression was higher in the LNM group than in the non-LNM group (P < 0.001) (Fig. 1C). Furthermore, the TNM III group showed a significantly higher Rab1A expression than the other group (P = 0.004) (Fig. 1D).

### Relationship between Rab1A and the clinic-pathological factors in GC patients

The relationship between Rab1A and clinic-pathological factors was analyzed using the Chi-square or Fisher's tests. The results are displayed in Table 1, indicating Rab1A expression was closely related to LNM (P < 0.001), degree of differentiation (P = 0.012), Venous invasion (P = 0.046), and TNM staging (P = 0.026). There was no significant difference between Rab1A expression and the left clinic-pathological factors.

**Table 1:** Relationship between Rab1A and clinic-pathological factors in 115 GC patients.

Variables	Rab1A		
	Negative	Positive	*P* value
Age (years)			
< 65	29	30	0.928
≥ 65	28	28	
Gender			
Male	38	42	0.503
Female	19	16	
Tumor size (cm)			
< 5	42	42	0.878
≥ 5	15	16	
Depth of tumor invasion			
T1–2	23	17	0.214
T3–4	34	41	
Lymph node metastasis			
No	31	11	< 0.001^c^
Yes	26	47	
Degree of differentiation			
Well	23	11	0.012^a^
Poor	34	47	
Venous invasion			
Negative	38	28	0.046^a^
Positive	19	30	
Neural invasion			
Negative	32	24	0.113
Positive	25	34	
TNM staging			
I–II	41	30	0.026^a^
III	16	28	

^a^*P* < 0.05, ^b^*P* < 0.01, ^c^*P* < 0.001.

#### The influence of Rab1A overexpression on prognosis in GC patients

The Kaplan–Meier analysis was used to explore the influence of Rab1A overexpression on prognosis. Our results showed that the Rab1A positive group had a worse prognosis than the negative group (P < 0.001) (Fig. 1E). Moreover, we performed subgroup analysis on prognosis depending on the various TNM states using the Kaplan–Meier analysis. Rab1A overexpression group showed a poorer 5-year survival rate than the low-expression group in TNM I–II staging (P < 0.001) (Fig. 1F). However, no significant difference was observed in TNM III staging (P = 0.222) (Fig. 1G). Univariate analysis indicated that differentiation degree, venous invasion, neural invasion, depth of tumor invasion, LNM, TNM stage, and Rab1A expression were closely related to poor prognosis (P < 0.05) (Table 2). Moreover, multivariate analysis showed that Rab1A expression, TNM stage, LNM, and neural invasion were critical prognostic factors in GC (Table 2).

**Table 2:** Results of univariate and multivariate analyses of postoperative patients’ survival by Cox’s proportional hazard model.

Varieties	Univariate analysis			Multivariate analysis		
	HR	95% CI	*P*	HR	95% CI	*P*
Age (≤ 60 or > 60 years)	0.931	0.563–1.479	0.711			
Gender (male/female)	1.349	0.777–2.345	0.288			
Size of tumor (≤ 5 or > 5 cm)	0.718	0.424–1.214	0.216			
Degree of differentiation (moderate-well/poor)	0.521	0.292–0.929	0.027^a^	0.745	0.407–1.362	0.339
Venous invasion (negative/positive)	0.424	0.260–0.691	0.001^b^	0.852	0.489–1.485	0.572
Neural invasion (negative / positive)	0.433	0.262–0.716	0.001^b^	0.647	0.362–1.164	0.147
Depth of tumor invasion (T1–2/T3–4)	0.360	0.201–0.645	0.001^b^	0.790	0.396–1.691	0.544
Lymph node metastasis (negative/positive)	0.265	0.146–0.482	< 0.001^c^	0.555	0.258–1.196	0.133
TNM stage (I–II/III)	0.300	0.183–0.491	< 0.001^c^	0.608	0.319–1.160	0.131
Rab1A expression (negative/positive)	0.372	0.224–0.618	< 0.001^c^	0.587	0.338–1.018	0.058

^a^*P* < 0.05, ^b^*P* < 0.01, ^c^*P* < 0.001.

### Overexpression of IL-4Rα led to poor prognosis in various TNM staging

**Figure 2:**
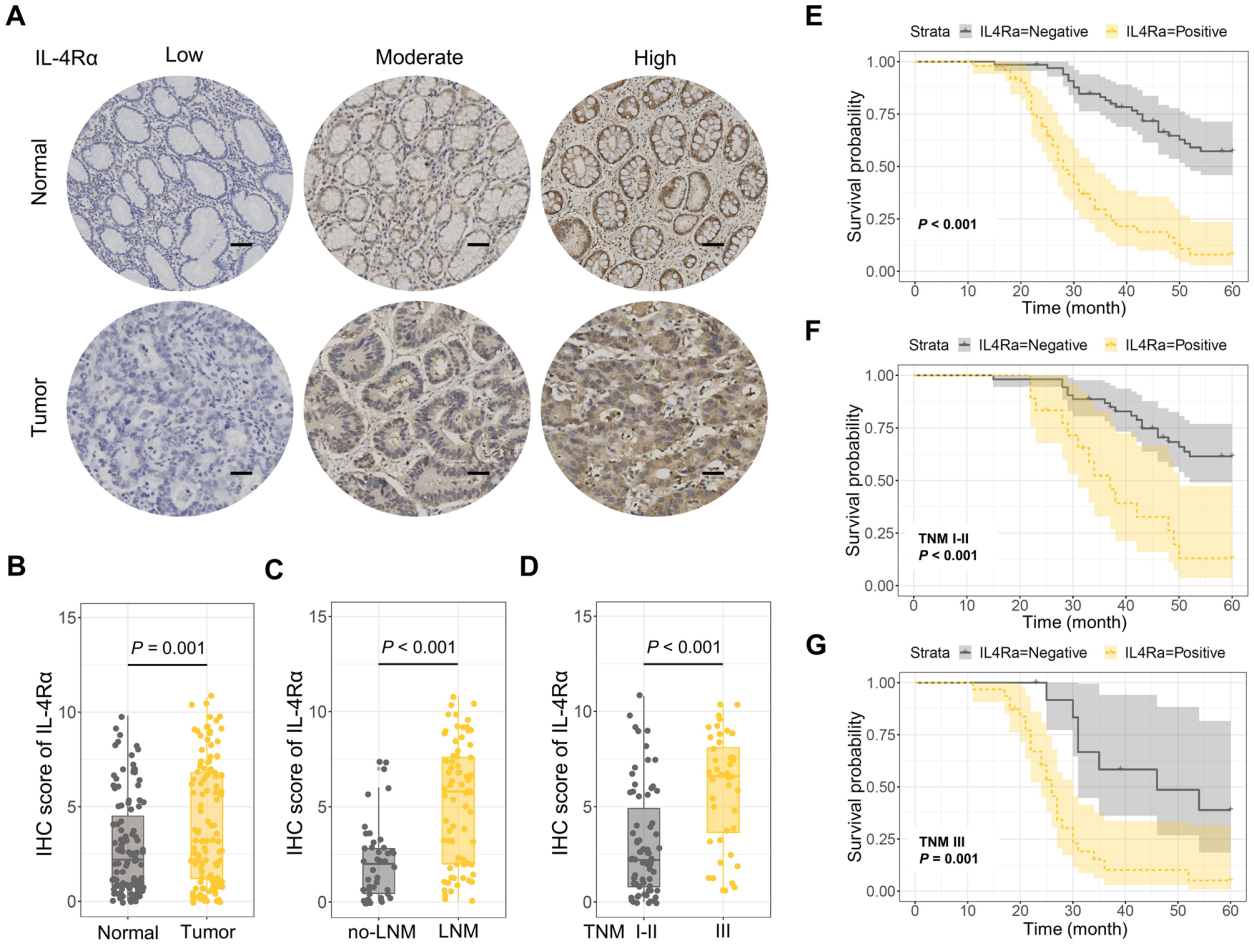
IL-4Rα expression and Kaplan–Meier analysis of 115 GC patients. (**A**) IL-4Rα expression using IHC staining in 115 GC and para-cancer tissues (scale bar 100um). (**B**–**D**) The IHC score of IL-4Rα in (**B**) GC and para-cancer tissues, (**C**) GC with or without lymph node metastasis, (**D**) GC with TNM I–II or TNM III staging. (**E**–**G**) Kaplan–Meier analysis of IL-4Rα positive vs. IL-4Rα negative in (**E**) 115 GC patients, (**F**) GC patients with TMN I–II staging, and (**G**) GC patients with TMN III staging.

The IHC staining was performed to investigate the IL-4Rα expression level in 115 GC patients to explore its influence on prognosis (Fig. 2A). IL-4Rα was significantly increased in GC tissues than in para-cancerous tissues (P < 0.001) (Fig. 2B). Moreover, subgroup analysis helped examine the IL-4Rα expression level according to different LNM and TNM staging. Thus, IL-4Rα expression in the LNM group was significantly higher than the non-LNM group (P < 0.001) (Fig. 2C). Additionally, the TNM III group showed higher IL-4Rα expression than the other group (P < 0.001) (Fig. 2D).

The Kaplan–Meier analysis indicated IL-4Rα overexpression caused poor prognosis (P < 0.001) (Fig. 2E). Moreover, subgroup analysis revealed that the group with increased IL-4Rα expression had shorter 5-year survival rates in TNM I–II and TNM III stages (P < 0.001, P = 0.001) (Fig. 2F,G).

### Positive correlation of Rab1A expression with the IL-4Rα expression in GC

**Figure 3:**
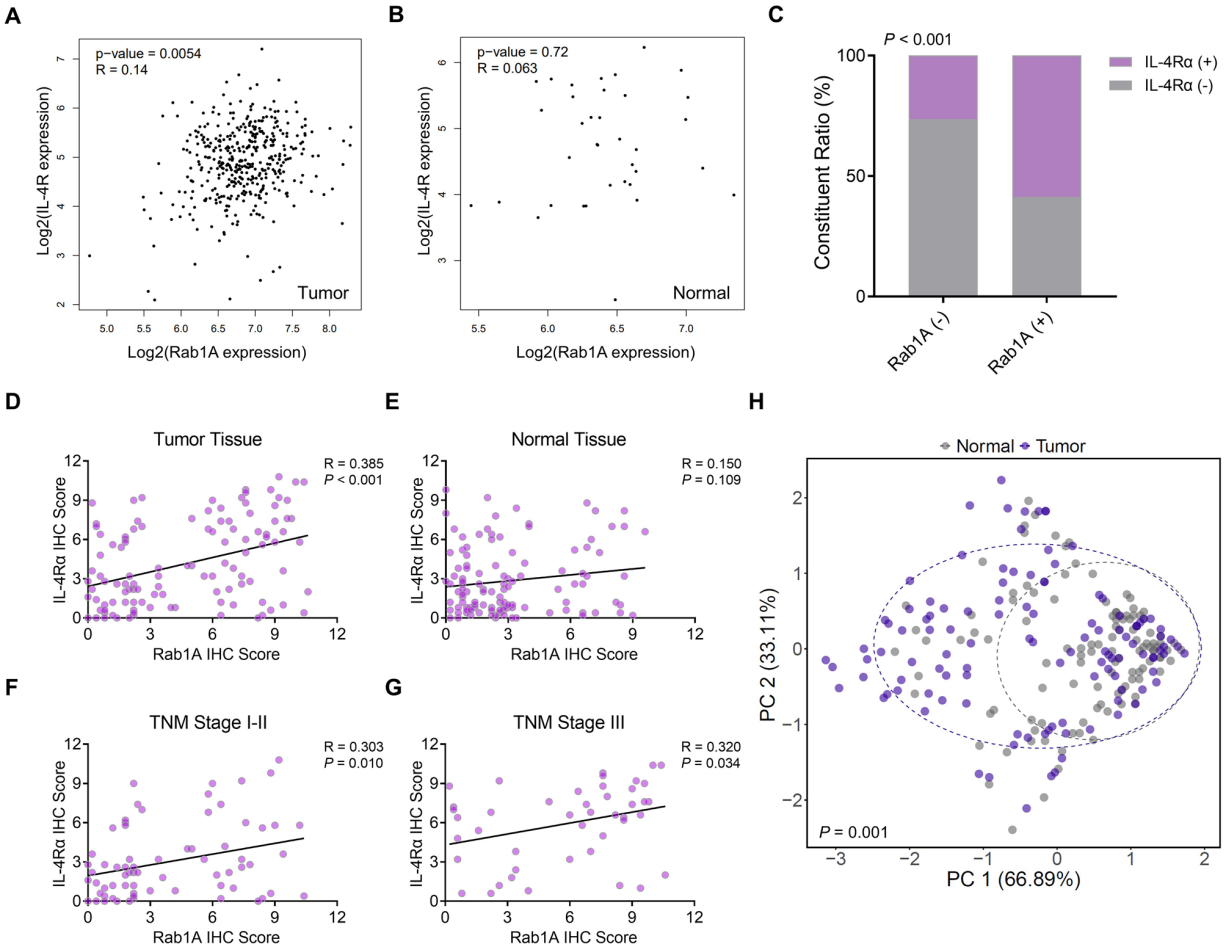
Association of Rab1A/IL-4Rα expression in GC. (**A**,**B**) the association between Rab1A/IL-4Rα expression in TCGA datasets through the GEPIA platform in GC tissues(**A**) and normal tissues (**B**). (**C**) Constituent ratio depicting the correlation between Rab1A and IL-4Rα expression in GC. (**D**–**G**) The association of Rab1A with IL-4Rα expression using the IHC score in (**D**) GC tissues, (**E**) normal tissue, (**F**) GC with stage TNM I–II staging, and (**G**) GC with TNM III staging. (**H**) Cluster analysis of Rab1A and IL-4Rα IHC score based on GC tumor and paired normal tissues.

We searched the TCGA database through the GEPIA platform to investigate the association of Rab1A and IL-4Rα expression. The results indicated Rab1A expression was closely related to the L-4Rα expression in GC tissues. In contrast, no significant correlation was observed on expression in para-cancer tissues (P = 0.005) (Fig. 3A), (P = 0.72) (Fig. 3B). Interestingly, Rab1A was significantly associated with IL-4Rα expression (P < 0.001) (Fig. 3C). Then the IHC staining helped validate the association of Rab1A/ IL-4Rα in 115 GC patients. The outcome indicated that Rab1A expression was significantly associated with IL-4Rα expression in GC tissues (P < 0.001) (Fig. 3D). However, no significant difference could be found in para-cancer tissues (P = 0.109) (Fig. 3E). The subgroup analysis across various TNM stages had a positive correlation of Rab1A/IL-4Rα expression in TNM I–II and TNM III staging (P = 0.010, P = 0.034) (Fig. 3F,G).

Rab1A/ IL-4Rα expression was significantly elevated in GC tissues than in para-cancer. Moreover, Rab1A expression was mainly related to IL-4Rα expression in GC tissues. Thus, a cluster analysis was performed depending on the IHC scores of Rab1A/IL-4Rα in GC and normal gastric tissues (Fig. 3H). The PERMANOVA analysis revealed that the IHC scores of Rab1A and IL-4Rα significantly differed between tumor and normal tissues (P = 0.001). Therefore, Rab1A and IL-4Rα levels can be differentiated between GC and normal tissues.

**Figure 4:**
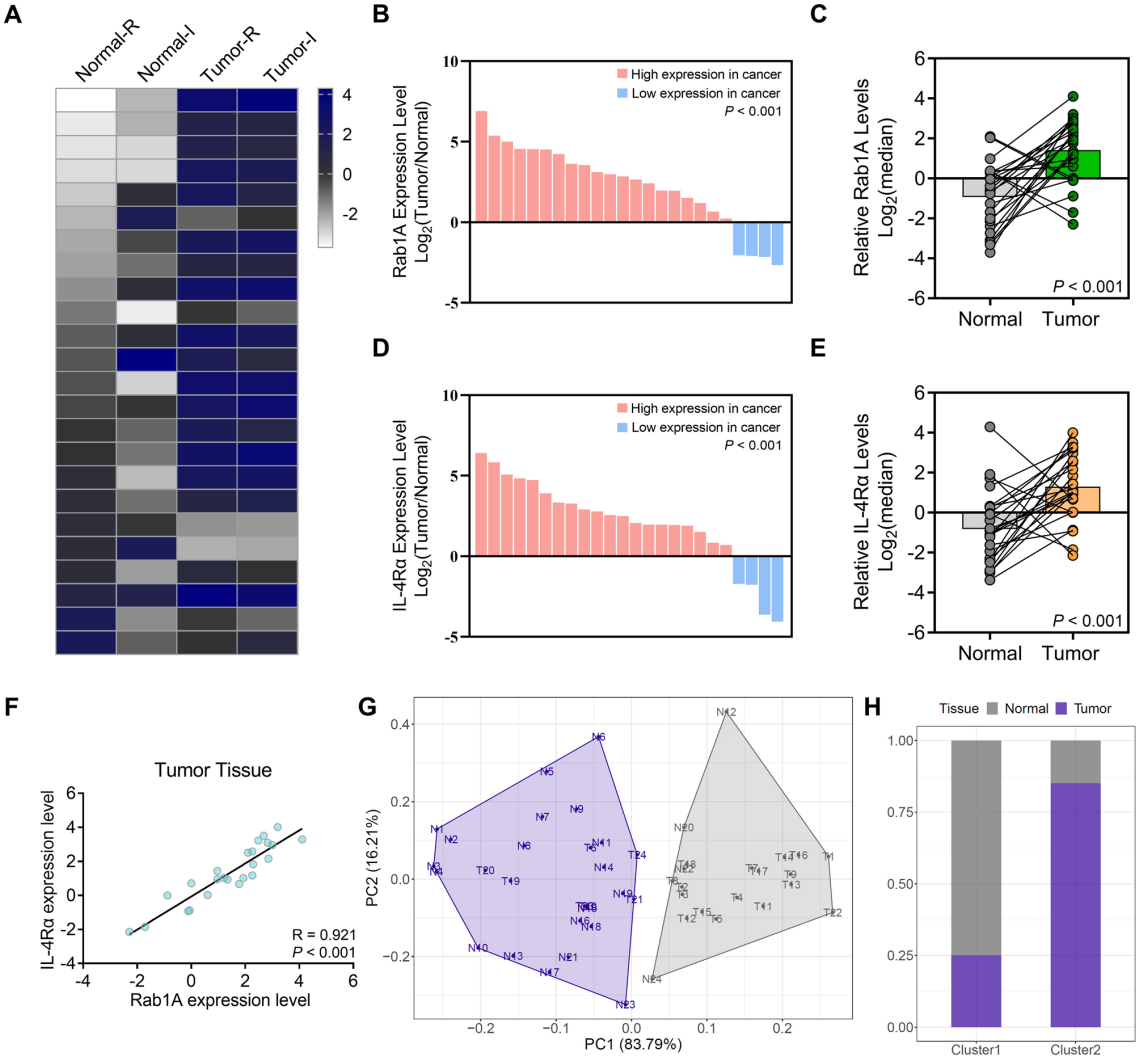
The correlation between Rab1A and IL-4Rα expression in 24 GC and paired normal tissues using *qPCR*. (**A**) the heatmap exhibits the correlation between Rab1A and IL-4Rα expression in GC and para-cancer normal tissues. (**B**,**C**) Rab1A expression in GC and para-cancer tissues. (**D**,**E**) IL-4Rα expression in GC and para-cancer tissues. (**F**) The correlation between Rab1A and IL-4Rα expression in 20 GC tissues. (**G**) Cluster analysis for normal and tumor tissues depending on the mRNA level of Rab1A/IL-4Rα in GC and normal gastric tissues and (**H**) percentage of tumor and normal tissues in each cluster.

Finally, the *qPCR* helped explore the Rab1A/IL-4Rα mRNA expression in 24 tumor and normal tissue cases, displaying heatmap (Fig. 4A). The results indicated that the expression of Rab1A/IL-4Rα mRNA was significantly elevated in GC tissues than in normal tissues (P < 0.001) (Fig. 4B–E). Additionally, the mRNA expression of Rab1A was closely associated with that of IL-4Rα in GC tumor tissues (P < 0.001) (Fig. 4F). Furthermore, cluster analysis based on the mRNA level of Rab1A/IL-4Rα in GC and normal gastric tissues were performed (Fig. 4G), showing 25% tumor and 75% normal tissues within Cluster 1 (Fig. 4H).

### The influence of Rab1A/IL-4Rα overexpression on GC patient prognosis

**Figure 5:**
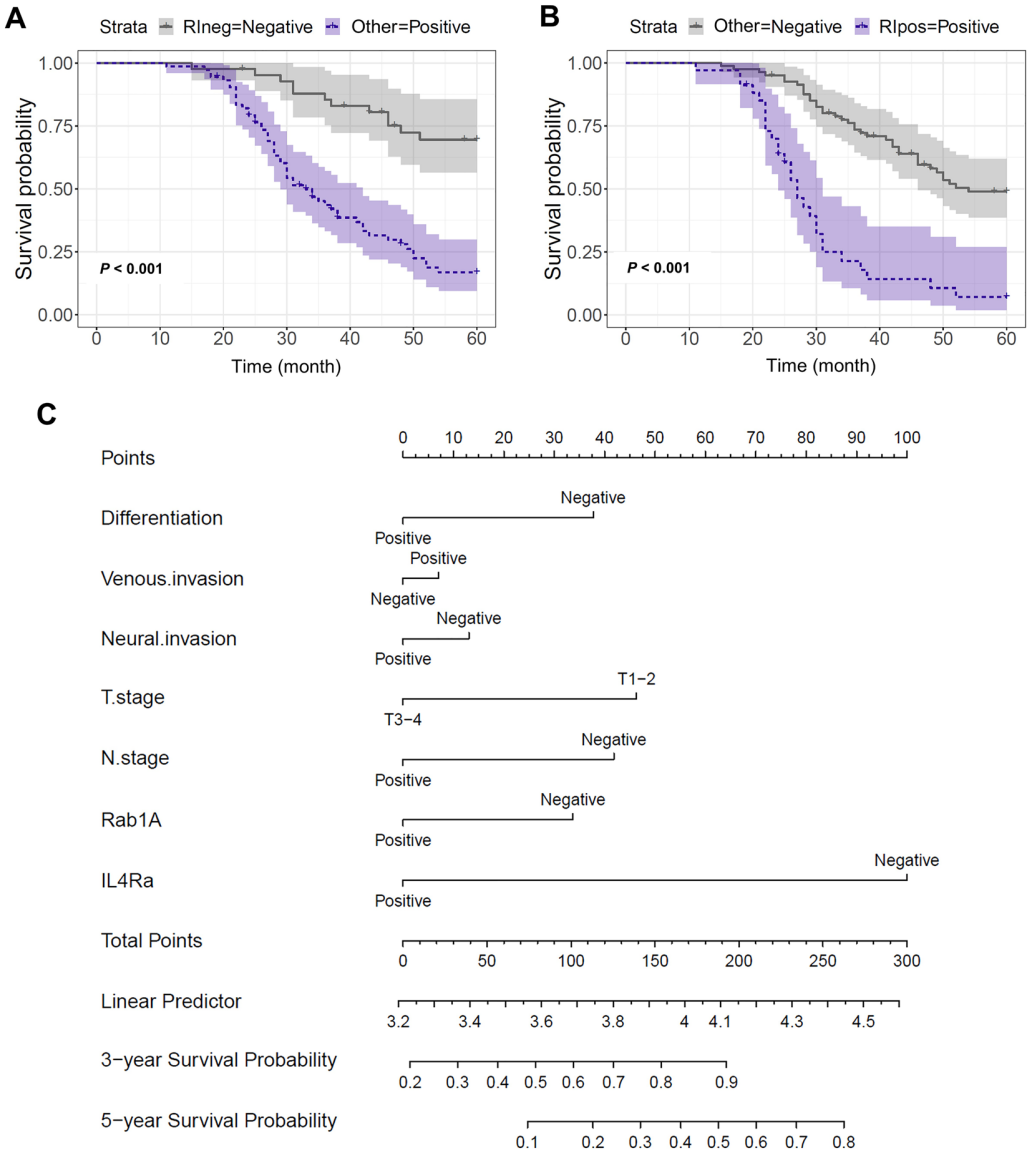
Nomograms predict 3 and 5-year overall survival of GC patients. The nomogram point scale helped predict the 3 and 5-year overall survival rates predicted using the total points associated with each prognostic factor.

Initially, the Kaplan–Meier analysis helped explore the influence of Rab1A/IL-4Rα overexpression on GC patient prognosis. Therefore, both negative expressions of Rab1A and IL-4Rα group showed longer 5-year survival rates than in the other group (P < 0.001) (Fig. 5A). Besides, positive Rab1A and IL-4Rα group expressions revealed a worse prognosis than the other group (P < 0.001) (Fig. 5B). Moreover, the nomograms helped predict the 3 and 5-year overall GC patient survival (Fig. 5C). The total points for each prognostic factor on the nomogram point scale predicted the survival rate. The outcome indicated Rab1A/IL-4Rα expression was crucial in predicting the 3 and 5-year overall GC patient survival.

## Discussion

GC is the fifth most common cancer worldwide and the fourth primary cause of cancer-related mortality^26,27^. Although the development of various diagnostic and treatment strategies for GC, the 5-year survival rate is < 30%. This is because most GC patients are diagnosed as an advanced stage or even have distant organ metastasis. Secondly, the resistance to chemotherapy drugs is also an important reason for the poor prognosis of patients with advanced GC^28^. Therefore, early diagnosis and finding novel therapeutic targets are urgent.

Rab1A is a RAB family member and a small GTPase studied frequently^7^. Rab1A is anchored to the endoplasmic reticulum and Golgi membrane while mainly expressing itself in the endoplasmic system^8,9^. Previous studies established that Rab1A was widely expressed in the ER with a deep exploration of Rab1A, increasing the pressure of the ER and promoting cell apoptosis^29^. Rab1A transports substances between membrane systems and is vital in transmitting transmembrane signals between cells^10^.

Recent studies indicated Rab1A, an oncogene, is aberrantly elevated in various cancers. Furthermore, Rab1A overexpression led to poor prognoses, including lung cancer^16^, liver cancer ^17^, and colorectal cancer^30^. A previous study reported that Rab1A and IL4Ra expression is highly associated with lung cancer tissues^25^. However, the potential correlation of Rab1A and IL4Rα remains largely unknown in GC. Our study mainly investigates the correlation between Rab1A and IL4Rα expression and the effect of Rab1A/IL4Rα on patient prognosis. This study first performed the IHC staining to assess Rab1A expression and determine the association between Rab1A expression and clinic-pathological factors. Rab1A was more highly expressed in GC tissues than in matched normal tissues. Furthermore, the subgroup analysis depicted that Rab1A expression in the LNM group was significantly higher than in the non-LNM group. Moreover, the TNM III group had a considerably higher Rab1A expression than the other group. Besides, the results indicated that Rab1A expression was closely associated with LNM. No significant difference was observed between Rab1A expression and tumor size or T staging. Thus, Rab1A could promote the ability to migrate.

A previous study has reported that Rab1A overexpression caused a poor prognosis in CRC^9,30,31^, GC^32^, and intrahepatic cholangiocarcinoma^19^. The Rab1A positive group had a worse prognosis than the negative group in GC patients. Moreover, the subgroup analysis revealed Rab1A overexpression group showed a poorer 5-year survival rate than the low-expression group in TNM I–II staging. Simultaneously, no significant difference was obtained in TNM III staging, indicating that Rab1A is crucial in prognosis, particularly in TMN I–II staging. In GC patients with TNM stage III, the prognosis is generally poor. Moreover, Rab1A expression cannot affect the prognosis of these patients.

A previous study reported that the expression of Rab1A and IL4Ra is highly correlated in lung cancer tissues, including the expression region and degree^25^. Our research first reported that IL-4Rα significantly increased in GC tissues than in para-cancerous tissues. Moreover, based on different LNM and TNM staging, subgroup analysis indicated that IL-4Rα expression in the LNM group was significantly higher than in the no-LNM group. The TNM III group showed a higher IL-4Rα expression than the other group. Then Kaplan–Meier analysis determined the effect of IL-4Rα overexpression on prognosis. The results indicated that IL-4Rα overexpression led to a poor prognosis. Moreover, subgroup analysis based on different TNM stages suggested that the group with high IL-4Rα expression led to shorter 5-year survival rates in TNM I–II and TNM III.

We first checked TCGA database using the GEPIA platform to assess the association of Rab1A and IL-4Rα expression. Rab1A expression was closely associated with the L-4Rα expression in GC tissues. In contrast, no significant correlation with expression was observed among para-cancer tissues. Then the IHC staining indicated that Rab1A expression was significantly associated with IL-4Rα expression in GC tissues. However, no significant difference could be seen in para-cancer tissues, consistent with the TGGA database. Finally, *qPCR* helped investigate the Rab1A/IL-4Rα mRNA expression in 24 tumor and normal tissue cases. Our results showed that the mRNA expression of Rab1A was closely related to the IL-4Rα mRNA expression in GC tumor tissues. Additionally, the Kaplan–Meier analysis helped explore the influence of Rab1A/IL-4Rα overexpression on GC patient prognosis. The results showed that both negative expressions of Rab1A and IL-4Rα group had longer 5-year survival rates than the other group. Besides, positive Rab1A and IL-4Rα group expressions had a worse prognosis than the other group. Finally, the nomograms predicting 3 and 5-year overall survival indicated that Rab1A/IL-4Rα expression had crucial roles in predicting the GC patient prognosis. Thus, the Rab1A/ IL-4Rα could predict patient prognosis and be tractable as novel targets in individualized GC therapy.

## Conclusion

In summary, Rab1A/IL-4Rα was significantly elevated in GC tissues than in para-cancerous tissues, and its overexpression caused poor prognosis in GC patients. Rab1A expression was significantly related to IL-4Rα expression in 115 GC tissues using the IHC staining score analysis. Besides, the Kaplan–Meier analysis revealed that the group with negative Rab1A and IL-4Rα expression had longer 5-year survival rates than the other group. Therefore, Rab1A/IL-4Rα is vital in GC, providing a novel vision for targeted GC therapy.

## Materials and methods

### Patients and tissue specimens

One hundred fifteen cases of GC and para-cancer tissues were obtained from 2015 to 2017 from the Department of General Surgery, the First Affiliated Hospital of Wannan Medical College. Twenty-four cases of fresh GC and adjacent normal tissues were recruited in 2022 from the same department. These GC patients had not undergone preoperative chemoradiotherapy before surgery. The study was approved by the Independent Ethics Committee (IEC) of the First Affiliated Hospital of Wannan Medical College (IRB number 202248). All the patients provided their written informed consent.

### Immunohistochemistry (IHC)

The immunohistochemical staining (IHC) helped detect the Rab1A/IL4Ra expression in 115 GC and para-cancer tissues. These GC tissues were fixed in formalin, embedded in paraffin, cut into 5um, and stained using IHC, based on our previous study ^32^. Sections were incubated for two hours using the anti-Rab1A and anti-IL4Ra at 1:100 dilution at room temperature. The process was visualized using the tissue staining kit (Zhongshan Biotechnology, Beijing, China). The staining score was calculated based on our previous study. Five regions were randomly selected for staining evaluation, and the IHC score was determined by multiplying the staining intensity (0, negative; 1, weak; 2, moderate; and 3, strong) and extent (0, 0–5%; 1, 6–25%; 2, 26–50%; 3, 51–75%; and 4, > 75%). We considered 0 as − ,1 ~ 4 as + , 5 ~ 8 as ++, and 9 ~ 12 as +++, for the final staining score. Our study regarded ++ or +++ as a high expression and – or + as a low expression. We used Anti-Rab1A (1:100, Abcam, ab302545) and anti-IL-4Rα (1:100, Abcam, ab203398) antibodies for immunohistochemistry.

### RNA isolation and quantitative real-time PCR (*qPCR*)

According to the manufacturer's protocol, we extracted total RNA from the 24 matched cases of fresh GC and adjacent normal tissue using TRIzol reagent (Invitrogen, Life Technologies, USA). Following the treatment of DNAse I (Thermo Fisher Scientific, USA) to remove genomic DNA, 1 μg RNA was reverse transcribed with a RevertAid First Strand cDNA Synthesis Kit (Thermo Fisher Scientific, USA). qRT-PCR was performed with Power SYBR^®^ Green PCR Master Mix (ABI, USA) on the 7500 real-time PCR system (ABI, USA) based on the manufacturer's instructions. We used the following primers: Rab1A sense (ACA GTG GCT GCA GGA AAT AGA) and antisense (AGC AAA TTC CTT CGC TGT TG); IL4Rα sense (CCG CCT CGT GGC TAT AAT AA) and antisense (CAG GGC AAG AGC TTG GTA AG). The 2^-ΔΔC^T method determined the fold changes relative to β-actin (internal control).

### Statistical analysis

The total data were expressed as means ± SEM. The statistical analyses were performed using the SPSS 22.0 software (SPSS Inc., Chicago, IL, USA), GraphPad Prism 8, and R program (version 3.6.1 for Windows, http://cran.r-project.org/). The t-test (unpaired, two-tailed) or Mann–Whitney U test helped compare the means between the groups. The Chi-square or Fisher's tests were used to explore the IHC results. Univariate and multivariate analyses were performed using SPSS 22.0 following relevant guidelines and regulations, with P < 0.05 being considered a significant difference. The authors have confirmed that all methods were carried out in accordance with relevant guidelines and regulations.

## Data Availability

The datasets used and/or analyzed during the current study available from the corresponding author on reasonable request.
